# Intelligence

**Published:** 1831-11

**Authors:** 


### INTELLIGENCE.

### Mr. Beaumont's *New Mode of Treating Fractures of the Leg and Fore-arm.*

In our last number we gave a notice of Mr. Beaumont's pamphlet: we have since seen the apparatus he employs: his mode of extending, and of keeping the fractured limb of a proper length, is very simple and ingenious. The principal objection we had to the proposed plan was the presumed difficulty of examining the state of the limb during the progress of the case; but Mr. B. has convinced us that this difficulty does not really exist, for any part of the plaster of Paris envelope can be easily removed without disturbing the limb, if a particular examination of it be required. We have received the following explanatory letter from Mr. Beaumont:

*To the Editor of the London Medical and Physical Journal.*

Sir: I beg leave to thank you for the very liberal critique which you have been pleased to pass on a pamphlet entitled "Observations and Experiments on a New Mode of Treating Fractures of the Leg and Fore-arm." I was not (as you have done me the favor to suppose,) acquainted with the Arabian mode of curing fractured limbs, which, though it is the same so far as regards the material employed, yet in other respects there is some essential difference, especially in the treatment of compound fractures; in which cases the Arabians use plaster of Paris only as a means of steadying and maintaining an unvarying position of the broken bone or bones: for as their method allows an exit to any blood, pus, or other fluid, which may have been shed or secreted by the injured parts, so their method must also allow a communication between those parts and the atmosphere. I have used plaster of Paris in compound fractures, not only for the purpose of maintaining an unremittingly motionless state, and a proper position of the broken bones, but also as a means of excluding with certainty the atmosphere from the injured parts; the great advantage of which I have stated. In the treatment, then, of compound fractures, there is a total difference of principle between the Arabian method and that which I have proposed.

The mode of applying the case of plaster is also (as you have observed) different: the Arabians pour plaster of Paris beneath the limb, so as at first, and by degrees, to make perfect this part of the incrustation. I at once incase the whole limb, with the exception of little else than about half a dozen spots (each not larger than a half-crown) on the under surface of it, which spots correspond with as many eminences, that serve, during the incrusting of the limb, as regular points of support, as a means of preventing any sinking of the limb in the situation of the fracture. It does not appear to me that, by the Arabian method, there can be any regular support to the limb during the deposition of the plaster beneath it; and where a fracture is very oblique, it is not stated that the Arabians employ any apparatus to make gradual and proper extension in the direction of the axis of the bones, or to maintain the limb so extended during the hardening of the plaster.

The Arabians make "deep incisions into the soft plaster, both lengthwise and across, though not quite through it:" by this procedure the incrustation must be very liable to be broken, should one attempt to change the position of the patient. But why make these incisions before they are needed? considering that it is quite as easy to make them (with a proper saw) when the plaster is dry and hard, as it is to make them when the plaster is soft. I am, therefore, sure that the Arabian method does not afford a greater facility of examining the limb during the progress of the cure than that which I have proposed: in either case, I believe that more time would be requisite for the examination, and the again making perfect of the incrustation, than surgeons could be expected frequently to bestow; but I also believe that an examination would very rarely be necessary.

If, as I fear, I may not have made myself clearly understood, it would give me great satisfaction to shew a cast of a limb, which I have put up in the manner I have proposed. I have the honour to be, sir, your very obedient servant,

WM. BEAUMONT.

8, *Fitzroy street, Fitzroy square;*
*October* 12*th*, 1831.

---

### FACTS REGARDING CHOLERA.

Great pains have been taken by Drs. Russel and Barry in tracing the history of cholera as it manifested itself at St. Petersburg and the adjacent places. Their inquiries have been chiefly directed to ascertaining its mode of propagation, with a view to determining the question of contagion; and although some of the facts are difficult to reconcile with any of our prevalent theories upon the subject, yet we must say that the great majority of them point to infection conveyed by persons, and not by goods or clothes, as the usual source of the contamination. The gentlemen alluded to seem to have exerted themselves to lay aside all preconceived opinions, marking the events passing under their own immediate observation, and in tracing the history of what they did not themselves witness; admitting only the testimony of those who had seen what they narrated; a caution which they were led to adopt in consequence of the colouring which they found to be often giving to circumstances according to the views entertained by the narrator as to the nature of the disease itself. Of the many important facts which their last letters (received a few days ago) contain, we can only give a few, but we hope soon to see the whole laid before the public in a systematic shape. There is at St. Petersburg a city prison, under the medical charge of Dr. Bish, an intelligent physician, and previously to the appearance of the epidemic, an anti-contagionist. He, as well as all the other officers of the establishment, resides within the walls of the prison, by which all communication with the neighbourhood is prevented. On cholera appearing at St. Petersburg, all intercourse between the gaol and the town was rigidly cut off, and no instance of the malady occurred for some time; but at length the wife of one of the prisoners, she being also a prisoner, was readmitted from one of the hospitals to which she had been sent to be cured of a syphilitic complaint, no one labouring under such being retained for treatment in the prison. On her return, she passed through the apartment where her husband was: she spoke to, and saluted him, but proceeded in a moment to the part of the building appropriated to females. She had some diarrhœa when admitted, which on the following night proved to be cholera, and of which she died in twelve hours. Three women, who had been in the room with her, were next taken ill, and after them her husband, who also died. There were four hundred persons within the walls, and of these twenty-seven had cholera, which in fifteen proved fatal. No case occurred in the portion of the prison allotted to nobles, it being apart from the rest.

There is a German colony on the Neva, thirteen versts from St. Petersburg. The houses are detached with gardens, and the surrounding country highly cultivated, while the inhabitants are more cleanly, and fonder of the open air, than the Russians. Thither some persons fled on cholera appearing in the capital; one of these, a female, took the disease, and died of it; but it did not spread, no other instance of it having occurred, though her bed seems afterwards to have been used. Indeed, there are many instances in which the beds and clothes of those who have died seem to have been made use of with impunity. Opposite this colony, on the other bank of the river, which is there about the breadth of the Thames at Blackfriars, are Russian villages; but they did not escape, though the locality was as salubrious as that of their German neighbours. It is a curious fact, that another German colony, on the road to Moscow, between Yshora and Colpina, escaped, although the disease raged at both the places just mentioned; but it was observed that no travellers ever stopped at the German village, because the others afforded much better accommodation.

At Cronstadt, as at Moscow, no case occurred among the military cadets (150 in number), all communication having been cut off between them and the rest of the fortress; but, on the other hand, several instances happened where persons had the disease, though no communication with any source of infection could be traced. Thus a man, confined by hæmoptisis on the third floor of a house, who had not been out of the room for many days, and who saw no one who had been exposed to the disease, took it notwithstanding. At the Foundling Hospital a good many children died of cholera, and several nurses had it; and it is a curious fact, that when any of these last who were suckling had the disease, so as to render it necessary for the infant to be given to another nurse, none of these who gave the breast in this way became affected with cholera, although, in many instances, the infant's clothes were not changed.

*Quarantine.* We understand that most vigorous arrangements have been made for the purpose of excluding cholera from our shores, if this is to be effected by quarantine. We hear that a considerable additional number of ships of war have, within the last week, been ordered upon this service.

Drs. Russel and Barry. The Russian government has conferred the decoration of St. Anne, of the second order, on Drs. Russel and Barry, for their zealous and scientific investigations on the subject of cholera at St. Petersburg. These gentlemen are supposed to be now at Hamburgh, where the presence of the cholera will probably detain them for some time.

*The Cholera at Vienna.* The mortality from the cholera in this capital, during the first month of its visitation, that is to say, from the 13th of August to the 13th of September, amounted to no more than one hundred deaths: but, about the latter period, a heavy rain and storm having prevailed for the better part of three days, the disease broke out with the most alarming violence on the night of the 13th, and in the course of four-and-twenty hours, swept away eighty victims; and this principally in the city. In one little street, not containing above ten or a dozen houses, six persons died on that night. The higher classes seem to have been peculiarly singled out for the ravage; and the Doctors Sidprowitsch, Gassner, and Roehrig, were among the first who fell. Since this alarming burst, however, the cholera has not gone on with proportional fury: it is comparatively tranquil, though the deaths are still by no means inconsiderable.

If the local peculiarities of Vienna be taken into account, it is not difficult to explain why the higher orders have particularly felt the severity of the disease, and also why the city has been its principal haunt. Vienna, the city, is small, compact, and surrounded by suburbs, from which it is separated by a wide glacis. The streets are very narrow, and the houses immoderately high. Yet it is here that the higher classes reside, though their first-floors are generally destitute of both air and light. The lower classes, meantime, enjoy the upper stories, as well as the more spacious and airy suburbs.

It should not escape observation, too, that Vienna is almost completely hemmed in with a chain of mountains; which, however, do not protect it from violent gales during the best part of the year, and especially about the equinoxes.

Rheumatism, in a severe form, is a constant resident at Vienna; and, every autumn, a malignant dysentery prevails, of which the mortality is considerable. The people, too, it may be remarked, particularly the higher orders, are fond of good living.

On the whole, it would seem to augur well for the future progress of the complaint, that in Vienna it has not been more severe; for few cities in Europe, perhaps, possess more of those elements which constitute the fomites of this direful malady.—*Med. Gazette.*

### METEOROLOGICAL JOURNAL,

By Messrs. Harris and Co. Mathematical Instrument Makers, 50, High Holborn.

| | Moon. | Rain gauge. | Thermom | | | Barometer. | | De Luc's Hygrometer. | | Winds. | | Atmospheric Variation. | | |
|---|---|---|---|---|---|---|---|---|---|---|---|---|---|---|
| Sept. | | | 9 a.m. | max. | min. | 9 a.m. | 10 p.m | 9 a.m. | 10p.m | 9 a.m. | 10p.m. | 9 a. m. | 2 p.m. | 10 p.m. |
| 20 | | | 61 | 65 | 51 | 29.64 | 29.65 | 62 | 62 | WNW | SSW | Fine | Fine | Cloudy |
| 21 | ○ | | 57 | 62 | 54 | .60 | .63 | 59 | 58 | WSW | S | Rain | Cloudy | Fine |
| 22 | | | 58 | 65 | 50 | .74 | .78 | 56 | 55 | SSW | WNW | Cloudy | Fine | — |
| 23 | | .29 | 54 | 64 | 53 | .92 | .95 | 56 | 56 | W | W | Fine | — | — |
| 24 | | | 60 | 67 | 57 | 30.02 | 29.93 | 60 | 55 | WSW | SW | — | — | — |
| 25 | | | 62 | 67 | 56 | .86 | .80 | 58 | 59 | SW | SW | — | — | — |
| 26 | | | 59 | 66 | 58 | 29.77 | .76 | 60 | 61 | NNW | SE | Sleet | Cloudy | Fine |
| 27 | | | 61 | 65 | 57 | .71 | .64 | 63 | 61 | SSW | SW | Sho'ry | Fine | Cloudy |
| 28 | ☾ | | 60 | 64 | 57 | .55 | .52 | 63 | 65 | SE | SE | Fine | Cloudy | Rain |
| 29 | | .180 | 63 | 68 | 59 | .42 | .37 | 64 | 60 | SE | SE | Cloudy | Fine | Cloudy |
| 30 | | | 64 | 69 | 60 | .37 | .21 | 60 | 64 | SE | ESE | Fine | — | Rain |
| Octob. | | | | | | | | | | | | | | |
| 1 | | | 63 | 66 | 53 | .16 | .23 | 63 | 57 | SSW | ESE | Cloudy | — | Rain |
| 2 | | | 58 | 65 | 56 | .34 | .46 | 60 | 62 | ENE | SW | Cloudy | Fine | Cloudy |
| 3 | | .20 | 64 | 65 | 57 | .51 | .60 | 61 | 63 | SSW | SSW | Fine | Fine | Rain |
| 4 | | .25 | 60 | 63 | 55 | .64 | .74 | 61 | 61 | W | W | — | — | Cloudy |
| 5 | ● | .15 | 60 | 62 | 55 | .74 | .83 | 59 | 61 | W | SW | Cloudy | Fine | Fine |
| 6 | | | 62 | 65 | 60 | .74 | .74 | 62 | 64 | SW | SW | Cloudy | Sho'ry | — |
| 7 | | .10 | 65 | 68 | 59 | .53 | .50 | 63 | 62 | S | SW | Fine | Fine | — |
| 8 | | | 62 | 63 | 56 | .52 | .47 | 60 | 63 | SSW | W | Overc. | Rain | Fine |
| 9 | | | 60 | 65 | 54 | .48 | .51 | 64 | 60 | SW | S | Fine | Fine | Rain |
| 10 | | | 59 | 62 | 58 | .46 | .37 | 60 | 60 | SW | S | — | — | Cloudy |
| 11 | | .68 | 61 | 61 | 57 | .51 | .54 | 63 | 65 | SW | S | Cloudy | Rain | Cloudy |
| 12 | | | 58 | 60 | 55 | .56 | .44 | 65 | 66 | S | W | Rain | Cloudy | Fine |
| 13 | ☽ | .97 | 60 | 64 | 60 | .48 | .39 | 69 | 67 | SSW | S | Cloudy | Cloudy | Cloudy |
| 14 | | .05 | 62 | 64 | 58 | .42 | .48 | 63 | 62 | S *va.* | SW | Cloudy | — | — |
| 15 | | | 61 | 66 | 50 | .61 | .75 | 62 | 60 | WSW | WSW | Fine | Fine | Fine |
| 16 | | | 54 | 59 | 52 | 30.00 | 30.08 | 60 | 59 | WSW | WSW | — | — | — |
| 17 | | | 57 | 60 | 57 | .13 | .19 | 62 | 63 | WSW | W | — | — | — |
| 18 | | | 60 | 63 | 56 | .20 | .17 | 65 | 66 | NNE | SE | Cloudy | — | Cloudy |
| 19 | | .03 | 60 | 65 | 51 | .05 | 29.93 | 66 | 64 | SE | SE | Fine | — | Fine |

The quantity of Rain fallen, in the month of September, was 4 inches and 89.100ths.

